# Bias-Modified Schottky Barrier Height-Dependent Graphene/ReSe_2_ van der Waals Heterostructures for Excellent Photodetector and NO_2_ Gas Sensing Applications

**DOI:** 10.3390/nano12213713

**Published:** 2022-10-22

**Authors:** Ghazanfar Nazir, Adeela Rehman, Sajjad Hussain, Othman Hakami, Kwang Heo, Mohammed A. Amin, Muhammad Ikram, Supriya A. Patil, Muhammad Aizaz Ud Din

**Affiliations:** 1 Department of Nanotechnology and Advanced Materials Engineering, Sejong University, Seoul 05006, Korea; 2 Department of Mechanical Engineering, College of Engineering, Kyung Hee University, Yongin 17104, Korea; 3 Department of Chemistry, Faculty of Science, Jazan University, Jazan, Saudi Arabia; 4 Department of Chemistry, College of Science, Taif University, P.O. Box 11099, Taif 21944, Saudi Arabia; 5 Solar Cell Applications Research Lab, Department of Physics, Government College University Lahore, Lahore 54000, Punjab, Pakistan; 6 School of Materials and Energy, Southwest University, Chongqing 400715, China

**Keywords:** graphene, ReSe_2_, heterostructure, photodetector, NO_2_ gas sensor, Schottky barrier height

## Abstract

Herein, we reported a unique photo device consisting of monolayer graphene and a few-layer rhenium diselenide (ReSe_2_) heterojunction. The prepared Gr/ReSe_2_-HS demonstrated an excellent mobility of 380 cm^2^/Vs, current on/off ratio ~ 10^4^, photoresponsivity (R ~ 74 AW^−1^ @ 82 mW cm^−2^), detectivity (D^*^ ~ 1.25 × 10^11^ Jones), external quantum efficiency (EQE ~ 173%) and rapid photoresponse (rise/fall time ~ 75/3 µs) significantly higher to an individual ReSe_2_ device (mobility = 36 cm^2^ V^−1^s^−1^, Ion/Ioff ratio = 1.4 × 10^5^–1.8 × 10^5^, R = 11.2 AW^−1^, D* = 1.02 × 10^10^, EQE ~ 26.1%, rise/fall time = 2.37/5.03 s). Additionally, gate-bias dependent Schottky barrier height (SBH) estimation for individual ReSe_2_ (45 meV at V_bg_ = 40 V) and Gr/ReSe_2_-HS (9.02 meV at V_bg_ = 40 V) revealed a low value for the heterostructure, confirming dry transfer technique to be successful in fabricating an interfacial defects-free junction. In addition, HS is fully capable to demonstrate an excellent gas sensing response with rapid response/recovery time (39/126 s for NO_2_ at 200 ppb) and is operational at room temperature (26.85 °C). The proposed Gr/ReSe_2_-HS is capable of demonstrating excellent electro-optical, as well as gas sensing, performance simultaneously and, therefore, can be used as a building block to fabricate next-generation photodetectors and gas sensors.

## 1. Introduction

Graphene and other two-dimensional (2D) materials, particularly transition metal dichalcogenides (TMDs), have attracted much attention due to their unique electro-optical properties [1,2,3]. TMDs consist of smooth surfaces without any dangling bonds and possess significantly low surface states and trapping defects that cumulatively enable rapid charge speed and suppress charge scattering even in a few nanometer-thick layers [4]. Using these materials, the scientific community is able to develop many proof-of-concept devices such as field effect transistors (FETs) [5,6,7], photodetectors [8,9,10], supercapacitors [5,11], solar cells [12], gas sensors [13], electrochemical sensors [14] and biosensors [15,16,17], etc., for years. Amongst these, photodetectors and gas sensors are of significant interest as these possess the capacity of resolving energy and environmental concerns to a certain level [18]. Interestingly, in contrast to graphene (a zero bandgap material) [19], TMDs have finite bandgap values normally between 0.2 to 3 eV [20] depending upon the choice of material and its layer thickness and were found to be a potential substitute for traditional narrow bandgap materials for many electronic and optoelectronic applications. Moreover, their properties are strongly influenced by the choice of metal contacts (either ohmic or Schottky), energy band alignment, and types of TMDs [21]. Additionally, their electro-optical and gas sensor characteristics can also be modified by the electrostatic backgate voltage as well as channel region doping [22,23].

Furthermore, heterostructures (HS) fabricated on either TMDs or with other low-dimensional electronic materials have been found to demonstrate outstanding optoelectronic and gas sensing performance in comparison to their counterparts [24,25,26]. For instance, PbS quantum dots (PbS-QDs)/MoS_2_ heterostructure have shown a tremendously high photoresponsivity (~6 × 10^5^ AW^−1^) as compared to only an MoS_2_ photodetector. Such remarkable photoresponse properties are associated with strong light absorption characteristics of PbS-QDs [27]. Previously, we have demonstrated ZnO-QDs drop cast over MoS_2_ nanosheets to study their electro-optical characteristics. The calculated photoresponsivity was found to be 2 × 10^3^ AW^−1^ [28]. Despite excellent photoresponse, these photodetectors have demonstrated low response speeds (0.1–10 s). This limited performance is ascribed to low response rates as well as the environmental hazard nature (Zn or Pb leaching) of QDs [29]. For the development of next-generation optoelectronic devices, high responsivity and rapid photoresponse is a prerequisite. To circumvent this issue, HS based on graphene and 2D TMDs have been established to improve photoresponse speed as well as photoresponsivity. For instance, the graphene/WS_2_ photodetector exhibited a responsivity of ~950 AW^−1^ with a response time of 7.85 s [30]. In another study, graphene/MoTe_2_ demonstrated a responsivity of ~971 AW^−1^ with a response time of 78 msec [31]. Such reasonable response speed is attributed to graphene’s high carrier transport [32].

Moving onward, 2D-materials-based gas sensors have also been found to be of significant interest. From the literature, the gas sensing response strictly depends upon the surface-to-volume ratio (SVR) of the material [33]. In this perspective, graphene was believed to outperform conventional sensors as its atomically thin layered structure possesses ultimately high SVR [34]. However, in addition to SVR, other factors that can influence gas sensing response are semiconducting properties and the density of available reactive sites for the occurrence of redox reactions [35,36]. Since individual graphene layers have no bandgap, however, stacking graphene to other TMDs can resolve this problem as the semiconducting properties of TMDs can easily be modified by the electrostatic gate bias or exposure to light and therefore, gas sensing response can be modulated/improved. Several 2D materials such as MoS_2_, GaSe, GaS, hBN, WSe_2_, etc. were investigated for gas sensing however, there is not much literature available on graphene-based TMDs heterostructures used as gas sensors [13].

Among various TMDs out there, rhenium diselenide (ReSe_2_) has been found as an excellent 2D semiconducting material possessing a theoretically measured DFT-based direct narrow bandgap (~0.995 (bulk)–1.239 eV (monolayer)) [37] which is significantly lower than conventional TMDs [38]. Recently, Kim et al. [23] investigated HCl-mediated p-doping of ReSe_2_ and reported an improved photoresponsivity of 1.93 × 10^3^ AW^−1^ and photoresponse rise/decay time of 1.4/3.1 ms as compared to undoped ReSe_2_ (photoresponsivity = 79.99 AW^−1^, rise/decay time = 10.5 ms/291 ms). Bach et al. [39] have studied Gr/ReSe_2_ barristor devices, however, with limited photoresponsivity of 42 AW^−1^ and rise/decay time of 33.9/20.8 ms under a high laser wavelength of 656 nm with a light intensity of 189 mW/cm^2^. However, these reports demonstrated lower photoresponse time. Moreover, no gas-sensing performance was demonstrated in these devices. Therefore, it is of great interest to develop a heterostructure that can demonstrate good electro-optical and gas sensing characteristics simultaneously within a single device.

Herein, we have successfully developed a Gr/ReSe_2_ hybrid device that can demonstrate exceptional photodetector and gas sensing performance, simultaneously. In our device, graphene and ReSe_2_ flakes work as transport and light absorption layers, respectively. We have drawn a comparative analysis of electro-optical performance between individual ReSe_2_ and Gr/ReSe_2_ devices. The results indicate that the Gr/ReSe_2_ photodetector has considerable photoresponsivity (R ~ 74 AW^−1^ at 82 mW cm^−2^), detectivity (D^*^ ~ 1.25 × 10^11^ Jones) and a high photoresponse (rise/decay ~ 75/3 μs) as compared to an individual ReSe_2_ device (R = 11.2 AW^−1^, D* = 1.02 × 10^10^, rise/fall time = 2.37/5.03 s). Moreover, the photocurrent and photoresponsivity were calculated as a function of laser light intensity. Furthermore, Schottky barrier height (SBH) evaluation has revealed Gr/ReSe_2_ devices demonstrating low SBH (9.02 meV at V_bg_ = 40 V) which is the reason behind the high electro-optical performance of the HS. Finally, Gr/ReSe_2_-HS was tested for NO_2_ gas sensing (20–200 ppb). The exceptional performance of our devices is ascribed to high-quality graphene, a suitable choice of ReSe_2_ flake, residual-free PDMS stamp supported transfer technique and choice of metal electrodes that eventually reveal low SBH.

## 2. Experimental Detail

### 2.1. Device Fabrication

Here, a vertical heterostructure (HS) composed of mono-layer graphene and few-layer ReSe_2_ was prepared over SiO_2_ (300 nm)/p^+^-Si substrate. Figure S1 illustrates step-by-step fabrication detail about HS formation. Briefly, CVD-grown monolayer graphene was transferred over SiO_2_/Si substrate by employing the wet transfer method reported elsewhere [40]. As-transferred graphene layer was then patterned into a rectangular shape (hall bar) using photolithography and an oxygen plasma etching process. During O_2_ etching, graphene was treated by power (~50 W) for a few minutes to etch undesired graphene. To make a large pattern around the graphene hall bar, a second photolithography process was carried out after which the defined electrodes were filled by Cr/Au (5/30 nm) deposition. On completion of the deposition, the devices were left in acetone for several hours to accomplish the lift-off process.

On another substrate (SiO_2_ (300 nm)/p^+^-Si), we used the scotch-tape method to mechanically exfoliate the ReSe_2_ flake. An optical microscope was used to find a suitable ReSe_2_ flake of a few-layers thickness which was later transferred to a pre-patterned graphene hall bar using a PDMS stamp and micromanipulator. In the end, the e-beam lithography process followed by Cr/Au (8/120 nm) deposition and subsequent lift-off in acetone were conducted to make final electrical connections to Gr/ReSe_2_ HS. Additionally, a moderate temperature annealing process (200 °C for 4 h) was also carried out in a tube furnace under Ar/H_2_ (97.5%/2.5%) gas flow to improve adhesion between metal electrodes and flake surfaces.

### 2.2. Characterization

Raman analysis was examined using micro-Raman (Renishaw, Wotton-under-Edge, UK) for monolayer graphene, few-layer ReSe_2_, and Gr/ReSe_2_ HS. A laser wavelength (514 nm) of low-power intensity (511 μW) with a spot size of 0.7 μm was used to avoid any kind of structural deterioration due to the laser heating effect. Furthermore, to realize the exact thickness of graphene and ReSe_2_ flakes, atomic force microscopy (AFM; n-Tracer, NanoFocus, Oberhausen, Germany) in tapping mode was used.

### 2.3. Electrical and Electro-Optical Measurement

For electrical measurement, Keithley 2400 and Keithley 6485 K (Keithley Instruments, Inc., Cleveland, OH, USA) were used as source meter and picoammeter, respectively. The complete electrical measurement was performed at room temperature and under vacuum (10^−3^ Torr) conditions. Further, to estimate Schottky barrier height (SBH), the device’s electrical measurement was achieved in the low-temperature range (30–300 K) under high vacuum (10^−4^ to 10^−5^ Torr). Moreover, to study electro-optical measurement, the devices were tested using the same systems (Keithley 2400 and Keithley 6485 K) under a vacuum in the dark and under laser light irradiation (532 nm) of varying power intensity (82–820 mW cm^−2^).

### 2.4. NO_2_ Gas Sensing Measurement

To further test the Gr/ReSe_2_ heterostructure ability to sense NO_2_ gas, an experimental setup illustrated in Figure S5 was utilized. The desired concentration was achieved by mixing NO_2_ (2%) and N_2_ (98%) before injecting them into the chamber. For this purpose, a mass flow controller (MFC) was employed which can control concentration and maintain a total gas flow rate of around 1000 sccm throughout the experiment. The gas is injected inside the chamber in such a manner that it reaches the Gr/ReSe_2_ HS-based sensor device within a few seconds. Such an experimental arrangement is very effective as it can detect any environmental change quickly. All the measurement was conducted at room temperature (26.85 °C) under ambient atmospheric conditions. The device was also irradiated with light illumination of 532 nm with a power intensity of 310 mW cm^−2^ to enhance the gas sensing response of the heterostructure.

## 3. Results and Discussion

**Figure 1:**
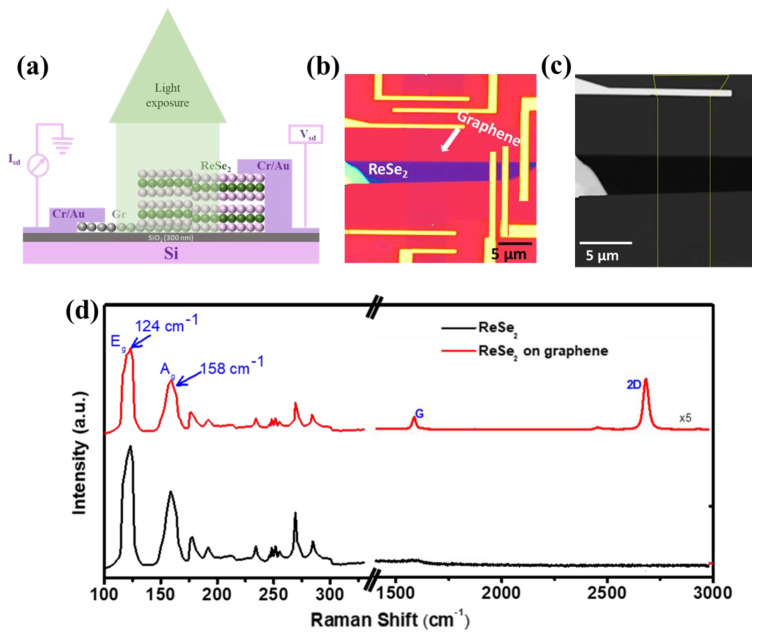
(**a**) Schematic illustration and (**b**) real device optical image of graphene/ReSe_2_ van der Waals heterostructure (scale bar; 5 µm), (**c**) SEM micrograph (scale bar; 5 µm), whereas yellow dotted line indicate graphene layer. (**d**) Raman analysis of ReSe_2_ (black curve) and graphene/ReSe_2_ heterostructure (red curve).

Figure 1a,b illustrates a schematic diagram and an actual device optical image of graphene/ReSe_2_ van der Waals heterostructure, respectively, fabricated at Si/SiO_2_ substrate and after Cr/Au contacts deposition via e-beam lithography process (scale bar: 5 µm). Figure S1 represents the device fabrication detail and the various steps involved. In brief, CVD-grown monolayer graphene was first etched into a rectangular bar after which a pristine ReSe_2_ flake of appropriate thickness was exfoliated over polydimethylsiloxane (PDMS) stamp, was transferred onto graphene and interacted through van der Waals forces using micromanipulation process. Of note, the photodetector devices based on individual ReSe_2_ and graphene/ReSe_2_ heterostructures have used the same ReSe_2_ flake to avoid any discrepancy while measuring photodetector device performance, as different ReSe_2_ flakes could have a different capacity for demonstrating photoresponse.

Further, Figure 1c represents a scanning electron microscope (SEM) micrograph (scale bar: 5 µm) which reveals a clear heterostructure without any deformation or contamination during the transfer process (*yellow dotted line indicates the boundary of monolayer graphene*). To further visualize the uniformity of the material and to evaluate the accurate thickness of the ReSe_2_ flake, the atomic force microscopy (AFM) image and corresponding height profile are presented in Figure S2. The AFM scanning reveals ReSe_2_ thickness to be around ~6.4 nm, approximately nine layers [41].

Raman analysis of ReSe_2_ and graphene/ReSe_2_ heterostructure have revealed several distinct peaks between the ranges 100–300 cm^−1^, ascribed to the interlayer vibrational decoupling in ReSe_2_ (Figure 1d). The prominent peaks related to ReSe_2_ were observed at 124 and 158 cm^−1^. These are associated with in-plane (E_g_) and out-of-plane (Ag) vibrational modes, respectively [42]. Furthermore, in the case of graphene/ReSe_2_ heterostructure, some additional peaks related to monolayer graphene were observed; G-peak positioned at 1580 cm^−1^ and is related to in-plane phonon mode, and 2D-peak located at ~2700 cm^−1^ is ascribed to double resonance [39,43].

### 3.1. ReSe_2_ Device Electrical Performance

**Figure 2:**
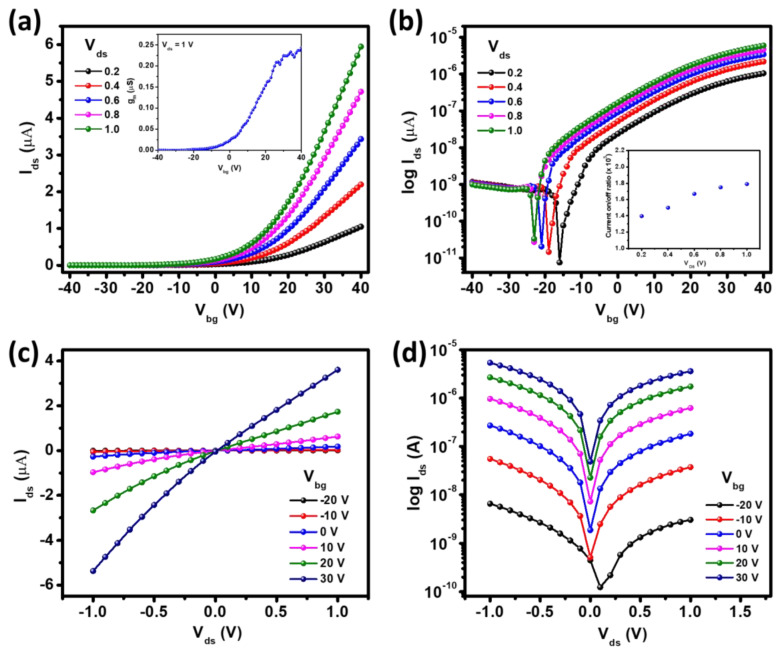
Transfer characteristics (I_ds_-V_bg_) of ReSe_2_ at V_ds_ = 0.2–1.0 V (**a**) in linear scale, and (**b**) in log scale (inset: current on/off ratio measured at V_ds_ = 0.2–1.0 V)). Output characteristics (I_ds_–V_ds_) in V_bg_ range from −20 to 30 V (**c**) in linear scale, and (**d**) in log scale.

To explore the device’s performance and to investigate the advantage of graphene in the heterostructure, the device’s electrical properties were realized from both pristine ReSe_2_ channel and Gr/ReSe_2_ heterostructure. Figure 2 illustrates the electrical performance of a few-layer ReSe_2_ flake fabricated on a Si/SiO_2_ substrate. The transfer characteristics (I_ds_–V_bg_) were studied at V_ds_ = 0.2–1 V and are presented both in linear-, and log-scale as shown in Figure 2a,b, respectively. A bias-dependent increase in on-current (I_on_) has been observed on increasing V_ds_ from 0.2 to 1 V which demonstrates usual ReSe_2_ transistor characteristics, similar to work [22,44]. The field-effect mobility denoted as µ_FE_ can be evaluated by the following relation:

(1) $${\text{µ}}_{\mathrm{FE}}=\frac{L}{W}\left(\frac{{\mathrm{dI}}_{\mathrm{ds}}}{{\mathrm{dV}}_{\mathrm{bg}}}\right)\frac{1}{C_{\mathrm{bg}}V_{\mathrm{ds}}}$$

In the above relation, the letters “L” and “W” indicate the length and width of the ReSe_2_ channel, $\frac{{\mathrm{dI}}_{\mathrm{ds}}}{{\mathrm{dV}}_{\mathrm{bg}}}$ denotes slope related to transfer characteristics, and $C_{\mathrm{bg}}$ (115  aF/μm^2^) represents gate capacitance [45,46,47]. The calculated mobility for the ReSe_2_ transistor was 36 cm^2^/Vs. In addition, the V_bg_-dependent trend of transconductance (g_m_(µS) = $\frac{{\mathrm{dI}}_{\mathrm{ds}}}{{\mathrm{dV}}_{\mathrm{bg}}}$) of ReSe_2_-based FET was presented (inset: Figure 2a) which demonstrate that the proposed devices possess promising potential of delivering larger gain. Furthermore, to find the suitability of the prepared ReSe_2_ transistors for digital applications, the devices must possess a current on/off ratio (I_on_/I_off_) of at least 10^4^ [48]. Figure 2b presents log-scale I_ds_–V_bg_ characteristics and the corresponding calculated I_on_/I_off_ ratio as the inset. Interestingly, the calculated I_on_/I_off_ ratio (~1.4 × 10^5^–1.8 × 10^5^) demonstrates an increasing trend with V_ds_ possibly due to an increase in on-current as observed in I_ds_–V_bg_ characteristics. The calculated mobility and I_on_/I_off_ are reasonably higher than the minimum requirement and surpass most of the previously reported TMDs on the Si/SiO_2_ substrate. Moving further, the output characteristics (I_ds_–V_ds_) related to the ReSe_2_ transistor were evaluated, as presented in linear scale (Figure 2c) and log-scale (Figure 2d), respectively. Almost linear I_ds_–V_ds_ characteristics in the low bias (±V_ds_) region reveal that the Cr/Au contact established nearly ohmic contact with the ReSe_2_ channel with low Schottky barrier height (see Section 3.3), as observed previously [49].

**Figure 3:**
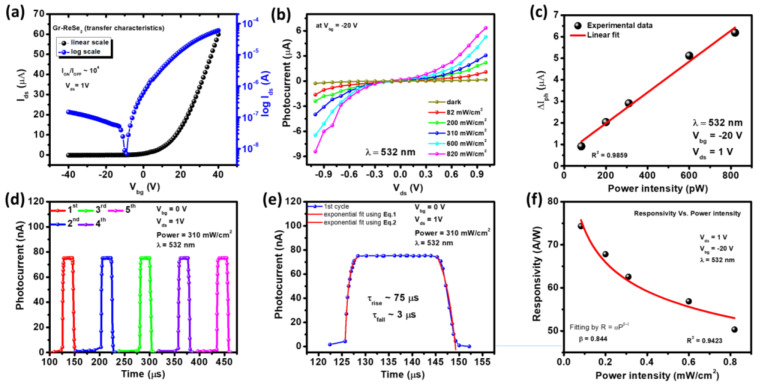
Graphene/ReSe_2_ heterostructure characteristics: (**a**) transfer characteristics (I_ds_–V_bg_) both in linear (black curve) and log-scale (blue curve) measured at V_ds_ = 1 V showing on/off ratio of ~10^4^. (**b**) Output characteristics (I_ds_–V_ds_) in dark and under various light intensities (82–820 mW/cm^2^) conditions measured at V_bg_ = −20 V and incident light wavelength of 532 nm. (**c**) Observation of linear relationship between ΔI_ph_ = I_ph_ – I_dark_ and light power intensities evaluated at V_bg_ = −20 V, V_ds_ = 1 V, and λ = 532 nm (where R^2^ = 0.9859). (**d**) Measurement of photocurrent for five consecutive cycles without any biasing at V_ds_ = 1V, power = 310 mW/cm^2^, and λ = 532 nm. (**e**) Corresponding measurement of photoresponse (rise time (75 µs) and fall time (3 µs)) of the graphene/ReSe_2_ heterostructure-based photodetector. (**f**) Responsivity versus power intensity trend follows relationship R = αP^β−1^ with calculated β = 0.844.

After the electrical transport measurement of the ReSe_2_ transistor, a detailed transport measurement was carried out to evaluate Gr/ReSe_2_ heterostructure electro-optical performance. Figure 3a illustrates the transfer characteristics (I_ds_–V_bg_) of Gr/ReSe_2_ van der Waals heterostructure both in linear-, and log-scale at V_ds_ = 1 V. It is noteworthy that the heterostructure demonstrates similar transfer characteristics as of pristine ReSe_2_ transistor (Figure 2a); however, a high on-current was observed in the heterostructure as compared to pristine ReSe_2_. This is ascribed to the higher carrier mobility of graphene [24]. The detailed transport characteristics (transfer and output) of pristine graphene are also presented in Figure S3. A charge-neutral point (CNP) also known as a Dirac point (DP) was observed around −8 V at V_ds_ = 0.1 V (Figure S3a), which indicates the graphene is a kind of n-doped graphene [50]. It should be noted here that no intentional doping was performed during the synthesis or transfer process. Therefore, the present monolayer graphene is regarded as pristine graphene. Further, the mobility was calculated using the relation *μ* = (1/*C_b_*_g_) (∂*σ*/∂*V_b_*_g_), where *σ* = 1/*ρ* represents sample conductivity. The measured value of electron mobility for monolayer graphene was around 1350 cm^2^/Vs. In addition, the output characteristics (I_ds_–V_ds_) were also performed (Figure S3b) which shows a linear relation, revealing the ohmic nature of Cr/Au contact with monolayer graphene. Such remarkable performance of monolayer graphene is the key reason for the high-performing Gr/ReSe_2_ heterostructure where we have observed mobility of 380 cm^2^/Vs and an on/off ratio ~ 10^4^). Here, Gr/ReSe_2_ heterostructure was prepared using CVD-grown monolayer graphene over which an exfoliated ReSe_2_ flake was transferred. Such heterostructure was also reported previously, however with a limited I_on_/I_off_ ratio of ~10^2^ [39]. However, the present work has demonstrated an I_on_/I_off_ ratio (10^4^), revealing the potential of the studied heterostructure for switching applications. The limited I_on_/I_off_ could be ascribed to graphene’s semi-metallic nature where the Fermi level of graphene and the related work function varies with bias voltage, resulting in controlled carrier transportation across valence/conduction bands. Moreover, the defects during the growth process of graphene and impurities through the transfer process could also play a significant role in controlling device electro-optical performance [51].

### 3.2. Gr/ReSe_2_ Photodetector Response

**Figure 4:**
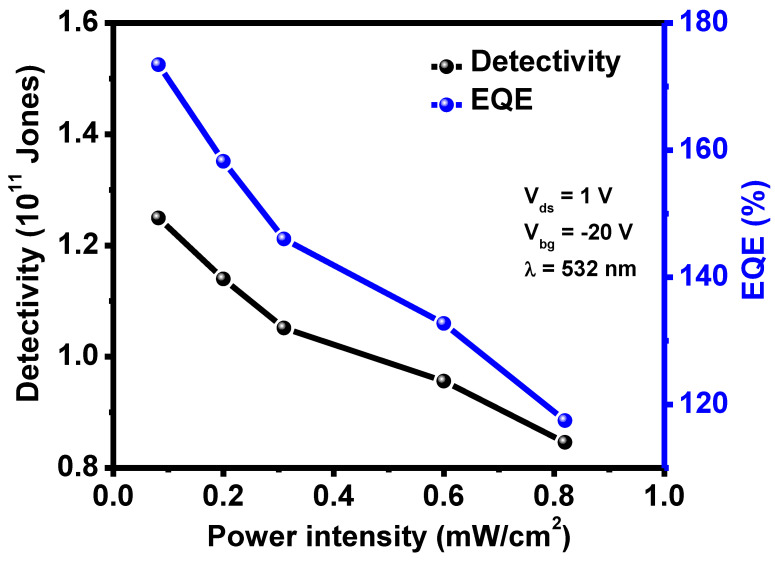
Calculation of detectivity (D*) and external quantum efficiency (EQE%) measured at V_ds_ = 1V, V_bg_ = −20 V, and λ = 532 nm.

To evaluate the photodetector performance based on Gr/ReSe_2_ heterostructure, photocurrent measurement as a function of V_ds_ at a fixed V_bg_ = −20 V and λ = 532 nm was presented in Figure 3b. The measured photocurrent at various incident light intensities is significantly higher than what has been observed under dark conditions, revealing the excellent photoresponse of active charge carriers inside the Gr/ReSe_2_ heterostructure. Interestingly, a linear relationship between ΔI_ph_ and light power intensities has been observed (Figure 3c) which indicates that the larger the light intensity, the higher will be the electron-hole pair generation which leads to the generation of high photocurrent in these devices [52]. Further, a cyclic measurement was performed which measured photocurrent for five consecutive cycles without any bias voltage at a power intensity of 310 mW/cm^2^, V_ds_ = 1 V and λ = 532 nm (Figure 3d). In this way, Gr/ReSe_2_ heterostructure photoresponse stability and results repeatability was verified. Noteworthy, the devices were measured under vacuum conditions to avoid external oxygen or water molecules device degradation. Moreover, the photoresponse was estimated at V_bg_ = 0 V to remove gate dependency or current contribution. However, the supplied V_ds_ was maintained at 1 V to facilitate drift to charge carriers in the channel region of the Gr/ReSe_2_ heterostructure. Furthermore, the photoresponse, i.e., the rise and fall time of the photodetector as a function of time, was estimated as shown in Figure 3e. The rise time (*τ_rise_*) and fall time (*τ_fall_*) of the photodetector was calculated using the following fitting equations [28]:

(2) Iph(t)=Idark+Aetτrise

(3) Iph(t)=Idark+Ae−tτfall

where “*I_ph_(t)*” represents time-dependent photocurrent, “*I_dark_*” indicate dark current under no light illumination, “*t*” denotes light switching time, and “*A*” is equation constant. Equations (2) and (3) were used to estimate the rise and fall time of the Gr/ReSe_2_-based photodetector. The calculated values for rise/fall time were 75/3 µs, significantly higher than most of the studied TMDs-based photodetectors [53,54,55]. We have also evaluated photoresponse characteristics from only the ReSe_2_ channel-based photodetector (Figure S4). The results indicate that Gr/ReSe_2_ photodetector has higher photo characteristics as compared to the ReSe_2_-based photodetector. In addition to the response time, several other important photodetector performance parameters such as photoresponsivity (R_λ_), external quantum efficiency (EQE%), and detectivity (D^*^) were evaluated and are presented in Figure 3f and Figure 4, respectively. The “*R_λ_*” is equal to photocurrent produced as a unit of light intensity incident on the effective channel area of the photodetector and is given by the relation [56]:

(4) $$R_{\lambda }=\frac{\Delta I_{Ph}}{PA}$$

where $\Delta I_{Ph}$ = $I_{Ph}-I_{dark}$ is the produced photocurrent, “*P*” denotes light intensity (82–310 mW/cm^2^) and “*A*” represents the device-effective area. The calculated *R_λ_* as a function of power intensity is presented in Figure 3f and has values between (50–75 A/W). Responsivity decreases as laser power increases. This trend was fitted by the equation $R_{\lambda }=\alpha P^{\beta -1}$ where α and *β* are constants whereas *P* corresponds to optical power. The calculated value of *β* was around 0.844 at maximum fit with R^2^ = 0.9423. The calculated value of $R_{\lambda }$ for Gr/ReSe_2_ photodetector is almost 7 times higher than ReSe_2_ photodetector (Responsivity ~11.2 AW^−1^).

*EQE* is the number of charge carriers produced per incident photon and mathematically expressed as [22]:

(5) $$EQE=\frac{hcR_{\lambda }}{e\lambda }$$

where, *h*, *c*, and *λ* are plank’s constant, speed of light and wavelength of the incident light, respectively. Interestingly, the *EQE* value is highly dependent on incident light wavelength, and for a fixed value of wavelength, it depends upon the value of photoresponsivity as other factors are constant. Figure 4 demonstrates a decreasing trend of *EQE* as a function of laser intensity and follows a similar trend as responsivity. The estimated *EQE* value was between 117–173% higher than the ReSe_2_ photodetector (*EQE* ~ 26.1%).

Detectivity (D^*^) is defined as the device’s ability to detect signals of a weaker strength. This is mathematically given by the relation [28,57]:

(6) D*=RλA122eIdark

D* is described in the unit of Jones, and one Jones = 1 cm Hz^1/2^ W^−1^ and *I_dark_* represent current under no illumination. Figure 4 shows D* as a function of light intensity follows a decreasing trend just like responsivity and *EQE*. It has values between 0.8–1.2 × 10^11^, significantly higher than ReSe_2_ photodetector (D* ~ 1.02 × 10^10^ Jones). All these results indicate Gr/ReSe_2_ van der Waals heterostructure-based photodetector has superior performance compared to only the ReSe_2_ material-based photodetector. This means graphene has a governing role in outperforming Gr/ReSe_2_ photodetector as it enhances transport rates of photo-carriers produced in ReSe_2_ due to the high carrier mobility provided by graphene. The photodetector performance was compared to previously published reports as presented in Table 1.

### 3.3. SBH Estimation

**Figure 5:**
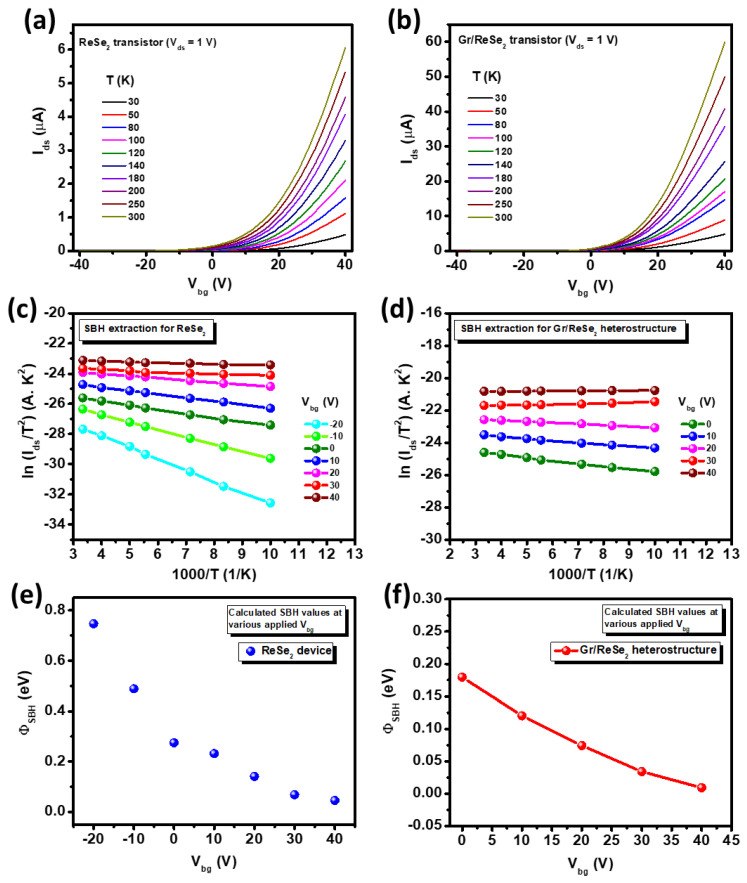
(**a**) Temperature-dependent transfer curves (*I*_ds_–*V*_g_) of ReSe_2_ and (**b**) Gr/ReSe_2_ transistors for temperature range 30–300 K and V_ds_ = 1V. No MIT evidence is visible. Richardson plot between ln (I_ds_/T^2^) and 1000/T at various V_bg_ for (**c**) ReSe_2_ device, and (**d**) Gr/ReSe_2_ heterostructure device. (**e**) Extracted SBH values versus V_bg_ for the ReSe_2_ device. (**f**) Extracted SBH values versus V_bg_ for Gr/ReSe_2_ device.

Next, we have estimated Schottky barrier height (SBH) denoted as Φ_SBH,_ and describe it as an energy barrier faced by the electrons while moving across the metal-semiconductor junction. Schottky–Mott’s rule was used to predict the value of Φ_SBH_. It states that the Φ_SBH_ varies proportionally with the difference between the semiconductor’s electron affinity and the metal’s work function. Interestingly, many semiconductors do not satisfy this rule due to the generation of metal-induced gap states which pin the bandgap close to the Fermi level. Such unwanted effect is regarded as Fermi-level pinning [58]. Therefore, it is highly desirable to select proper metal and semiconductors to minimize SBH value so that devices with ultimate electro-optical performance could be achieved. Here we chose Cr metal (work function ~ 4.5 eV) [59] to deposit as electrodes over the semiconductor (i.e., ReSe_2_ devices) and Gr/ReSe_2_ heterostructure to define the channel. Temperature-dependent transfer characteristics (I_ds_-V_bg_) for the ReSe_2_ transistor and Gr/ReSe_2_ heterostructure were determined and presented in Figure 5a,b. The curves were obtained at various temperatures (300, 250, 200, 180, 140, 120, 100, 80, 50 and 30 K). Noteworthy, in the transfer curve, the current values increase as the temperature increases contrary to previous reports which claim a kind of metal-to-insulator transition (MIT) around 200 K [22]. Since the devices prepared in the present work are realized over Si/SiO_2_ substrate, which possesses several impurities or defect states, it therefore hinders MIT observation in these devices. From the literature, it has been studied that gate-dependent carrier transport in thin layers of TMDs located near to dielectric substrate is strongly affected by the impurities and various disorders from the dielectric substrate. Therefore, these devices do not demonstrate MIT, which is in agreement with what we have observed in the present study. Moving further, the SBH value was calculated considering standard thermionic emission theory and using the below relationship [60]:

(7) $$I_{ds}=A_{area}A^{*}T^{2}exp\left(\frac{-q\Phi_{SBH}}{kT}\right)\left[exp\left(\frac{qV_{ds}}{\eta kT}\right)-1\right]$$

where $A_{area}$ represents the device’s effective area, A^*^ is Richardson’s constant, $I_{ds}$ source-drain current through the device channel, *V_ds_* indicate source-drain voltage, η is the ideality factor, *q* represents electron charge, *T* is temperature and k is the Boltzmann constants.

Figure 5c,d illustrates individual ReSe_2_ and Gr/ReSe_2_ heterostructure device’s Richardson plot, i.e., ln (I_s_/T^2^) versus q/kT in the reverse bias saturation regime where the obtained data was linearly fitted for each V_bg_ value. Based on the concept of thermionic emission theory, the slope of linearly fitted curves gives the value of Schottky barrier height (Φ_SBH_) as presented in Figure 5e,f. Interestingly, the calculated values of Φ_SBH_ are lower/higher at positive/negative V_bg_ values and do not vary linearly with the gate voltage. Moreover, there could exist three different transport regimes based on applied V_bg_ [61]. At low V_bg_, the device was considered in a switch-off state with the highest value of Φ_SBH_ and the only transport existed due to the thermal agitation of electrons crossing the barrier. Upon increasing V_bg_, the Φ_SBH_ decreases and the conduction band of ReSe_2_ started moving downward resulting in an exponential rise of current as obvious from the transfer characteristics of Figure 2b. Upon further increase in V_bg_, a flat band condition (V_bg_ = V_FB_) reaches which exists in the subthreshold region of transfer characteristics. Moving on, for V_bg_ > V_FB_, the device underwent a Schottky band regime as obvious by the bent downward part of I_ds_–V_bg_ characteristics, revealing a combination of thermionic and field emission transport. Finally, with more increase in V_bg_, there exist a tunneling current through Cr/ReSe_2_ barrier which became the major transport mechanism leading to the linear region in I_ds_–V_bg_ characteristics. As the devices are prepared over Si/SiO_2_ substrates, therefore, a lot of charge impurities and surface traps are expected from the substrate surface that could significantly affect the transport mechanism. It is interesting to note that SBH value is significantly lower for Gr/ReSe_2_ heterostructure (Φ_SBH_ = 179 − 9 meV for V_bg_ = 0–40 V) as compared to the individual ReSe_2_ device (Φ_SBH_ = 274 − 45.4 meV for V_bg_ = 0–40 V). Such low SBH value is dominated by thermionic field emission and could be attributed to the graphene layer which, in the case of Gr/ReSe_2_ heterostructure devices, acts as an impurity buffer layer. This will lead to a lesser amount of charge trapping in these devices which is evident from improved electro-optical performance in Gr/ReSe_2_ devices as compared to individual ReSe_2_ devices.

### 3.4. Energy Band Diagram

**Figure 6:**
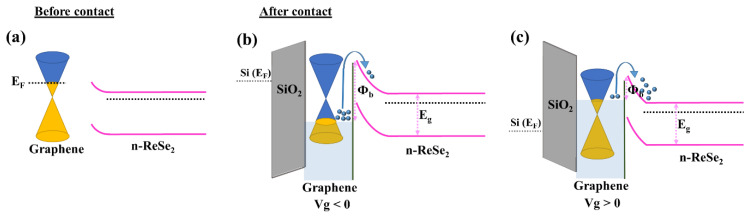
(**a**) Band diagram of graphene and ReSe_2_ before contact, (**b**) after contact when V_g_ < 0, and (**c**) after contact when V_g_ > 0.

To further understand, we have presented the energy band diagram of the Gr/ReSe_2_ heterostructure as shown in Figure 6a,b. Interestingly, the substrate impurities induce p-type doping of monolayer graphene leading to an increased density of holes within the graphene layer which, in turn, shift the Fermi level lower as compared to what was observed in the case of pristine graphene. Furthermore, from transfer characteristics (Figure 2), ReSe_2_ appears to be an n-type semiconductor; therefore, its Fermi level will be situated close to the conduction band. Moving further, as a result of ReSe_2_ transferred over CVD-grown monolayer graphene, a band bending occurs across the valence/conduction bands of ReSe_2_ to align the Fermi levels of both materials. This band bending is attributed to the work function difference between graphene and ReSe_2_. Upon biasing Gr/ReSe_2_ heterojunction, two types of band diagrams are possibly manifested in Figure 6b,c. It should be noted that the graphene layer is in direct contact with Si/SiO_2_ (300 nm) dielectric substrate; therefore, an externally applied electric field could significantly modify its Fermi level and thus the associated work function [62]. To calculate the Φ_SBH_ between graphene and ReSe_2_, a difference between graphene Fermi level and electron affinity of ReSe_2_ was obtained, i.e., Φ_SBH_ = Φ_Gr_ − χ_ReSe2_. From this relation, one can understand that Φ_SBH_ is strictly dependent upon Φ_Gr_ and can be modified if an external voltage is applied across graphene as it changes its work function in the heterostructure device. Figure 6b explains the band diagram under V_bg_ < 0 bias condition. In this state, graphene became more hole-doped as is obvious from the downward shift of the graphene Fermi level which eventually increases its work function and the Φ_SBH_. The value of Φ_SBH_ of Gr/ReSe_2_ heterostructure keeps on increasing with an increase in negative V_bg_. The highest value of Φ_SBH_ was observed at ~300 meV at V_bg_ = −40 V. Moving further, in the case of V_bg_ > 0, the electrons are generated in the graphene layer (Figure 6c). Under this condition, the Fermi level of graphene shifts in the upward direction resulting in a reduced Φ_SBH_ value. The lower value of Φ_SBH_ under forward biasing (V_bg_ > 0) facilitates easy transport of majority carriers across the junctions thus an increase in on-current was realized in the transfer characteristics (Figure 3a). As our heterostructure is composed of atomically thin flakes (i.e., monolayer graphene and few-layer ReSe_2_), the possibility of incomplete electric field screening in both materials cannot be evaded. Thus, both components of the heterostructure are affected by the electric field modulation, as evident in previous reports [63]. The estimated values provide an accurate assessment of Φ_SBH_ at the Gr/ReSe_2_ van der Waals interface via electric field modulation. Since the Φ_SBH_ demonstrates a strong gate modulation, one can speculate that the electric field-induced transport mechanism is the governing mechanism in the devices demonstrated here.

### 3.5. Gr/ReSe_2_ Heterostructure as NO_2_ Gas Sensor

**Figure 7:**
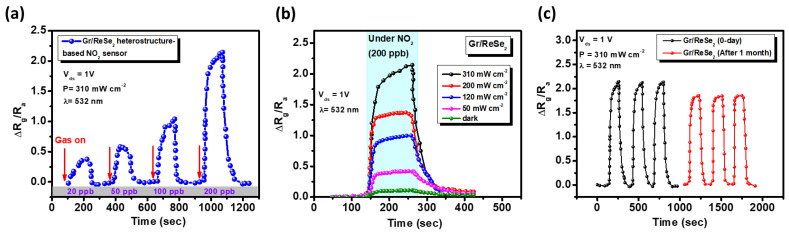
(**a**) Dynamic response of Gr/ReSe_2_ heterostructure under NO_2_ exposure with different concentrations at V_ds_ = 1V and laser light (532 nm) of power intensity 310 mW cm^−2^. (**b**) NO_2_ (200 ppb) gas sensing response of heterostructure under light wavelength (532 nm) exposure with increasing power intensity. (**c**) Heterostructure stability assessment under NO_2_ (200 ppb) with laser light (532 nm) intensity of 310 mW cm^−2^. The black curve (0-day) indicates the gas sensing performance of freshly prepared heterostructure, whereas the red curve demonstrates heterostructure performance after one month under ambient conditions.

To demonstrate the NO_2_ gas sensing experiment, the prepared devices were placed in a mass flow controller (MFC) setup as illustrated in Figure S5. Individual Gr, ReSe_2,_ and Gr/ReSe_2_ heterostructure devices were evaluated at room temperature under different gas concentrations (20–200 ppb). During the gas sensing experiment, the samples were continuously irradiated by a light illumination of 532 nm as it improves gas sensing response [26]. Additionally, the samples were irradiated by visible light instead of UV to avoid any damage from the light source. Figure 7a illustrates the gas sensing dynamic response of Gr/ReSe_2_ heterostructure under various NO_2_ concentrations at V_ds_ = 1 V and incident light illumination of 532 nm with the intensity of 310 mW cm^−2^. The gas sensing response was determined by the following relation:

(8) $$Response\left(\%\right)=\frac{\left|R_{g}-R_{a}\right|}{R_{a}}\times 100\%$$

where “*R_g_*” and “*R_a_*” indicate device resistance under NO_2_ gas environment and in air. Noteworthy, since two-dimensional materials (2D) bestow a large surface-to-volume ratio, their heterostructure could demonstrate a relatively high NO_2_ gas sensing response despite being under low NO_2_ concentration. It is obvious from Figure 7a that the Gr/ReSe_2_ heterostructure demonstrates a monotonically increasing gas sensing response with rising NO_2_ concentration from 20 to 200 ppb. Compared to previous reports on 2D materials-based gas sensors, our heterostructure demonstrates a large response of ~36% even at a low NO_2_ concentration of 20 ppb [64,65,66]. Moving further, we have explored our heterostructure gas sensing response for various light intensities. Under NO_2_ gas flow (200 ppb) and light wavelength (532 nm) exposure, the gas sensing response of heterostructure was evaluated with increasing light intensities as illustrated in Figure 7b. The gas sensing response rises from ~10% to ~200% as the light intensity increases from 0 to 310 mW cm^−2^. This is ascribed to the fact that more electrons are produced by increasing light intensity and made their way from the heterojunction to the NO_2_, subsequently leading to improved gas sensing response. Here, it is also noted that only Gr (black curve), and individual ReSe_2_ (blue curve) devices have demonstrated lower gas sensing performance of about ~20%, and ~41% as compared to Gr/ReSe_2_ heterostructure (~200%; red curve) under similar conditions as illustrated in Figure S6. This is attributed to enhanced electron-hole pairs generation at heterojunction interface under light exposure and agrees well with previous reports [67,68]. To further evaluate the NO_2_ gas response efficiency of the prepared Gr/ReSe_2_ heterostructure, the transient response was determined for 200 ppb NO_2_ concentration and under light illumination (532 nm) with intensity 310 mW cm^−2^ as displayed in Figure S7. As-calculated room temperature NO_2_ response/recovery time for the heterostructure was found to be 39/126 sec which is comparable with the top gas sensors based on 2D materials so far [69,70]. Next, the Gr/ReSe_2_ heterostructure was tested for gas sensing stability as illustrated in Figure 7c. The freshly prepared heterostructure (0-day) was placed under NO_2_ (200 ppb) and light wavelength (532 nm) with an intensity of 310 mW cm^−2^ exposure. The resultant NO_2_ gas sensing response was around ~200%. The device was tested again after a month under ambient conditions. The resultant gas sensing response was ~180% which is only 20% less than the original value, revealing the highly stable nature of Gr/ReSe_2_ heterojunction. Furthermore, to see the stable working potential of prepared sensor, relative humidity effect on response factor was tested as demonstrated in Figure S8. The results reveal similar sensing response under various humidity conditions, i.e., relative humidity (RH: 20–80%). The minor degradation was observed for RH = 20% and RH = 80%, however, no significant change was observed for RH: 40%, 60%, which is typical working conditions in most of the laborites. The results indicate that humidity is not the main factor for consideration to demonstrate consistent NO_2_ gas sensing behavior. Other parameters, such as NO_2_ gas exposure, light wavelength and intensity and exposure duration are the main factors that influence sensor properties.

**Table 1:** Comparison of photodetector performance of as-prepared FL-ReSe_2_ and Gr/ReSe_2_-HS with other reports from the literature.

Photodetector (Material)	Responsivity (AW^−1^) @ Wavelength	Internal or External Quantum Efficiency (%)	Detectivity (Jones)	Rise/Fall Time (s) OR Response Time	Refs.
Graphene	5 × 10^−4^	6–16%	-	-	[71]
Few layer ReS_2_	13 (220 nm)	0.73	-	6/21	[22]
1D Se–2D InSe heterojunction	3.2 × 10^−2^ (460 nm)	8.7	1.7 × 10^11^	3.0 × 10^−2^/3.7 × 10^−2^	[72]
BP/InSe	1.17 × 10^−2^ (455 nm)	3.2	-	2.4 × 10^−2^/3.2 × 10^−2^	[73]
Se-ReS_2_	36 (370 nm)	-	8 × 10^12^	<1 × 10^−2^/<1 × 10^−2^	[74]
CVD monolayer ReS_2_	13 (532 nm)	-	-	30-50 s	[75]
ReSe_2_/WSe_2_	0.28 (520 nm)	-	1.1 × 10^12^	4.7 × 10^−3^/4.1 × 10^−3^	[76]
ReS_2_/ReSe_2_	126.56 (350 nm)	-	1.76 × 10^11^	6.0 × 10^−6^/8.9 × 10^−6^	[77]
Sb_2_Se_3_/WS_2_	1.51 (520 nm)	-	1.16 × 10^10^	8.0 × 10^−3^/8.0 × 10^−3^	[78]
ReS_2_ bi-layer film	4 × 10^−3^ (500 nm)	0.99	-	10^3^	[79]
FL-ReSe_2_	11.2 (532 nm)	26.1	1.02 × 10^10^	2.37/5.03	**This work**
Gr/ReSe_2_-HS	74 (532 nm)	173	1.25 × 10^11^	75 × 10^−6^/3.0 × 10^−6^	**This work**

## 4. Conclusions

We have successfully fabricated Gr/ReSe_2_ van der Waals heterostructure (vdW-HS) using CVD-grown monolayer graphene (patterned into a rectangular bar), mechanically exfoliated few-layer ReSe_2_ and all-dry PDMS stamp-assisted transfer method. The prepared HS has been used to evaluate electro-optical properties and gas sensing performance. By exploiting narrow bandgap features of ReSe_2_, the prepared Gr/ReSe_2_-HS demonstrated an excellent mobility of 380 cm^2^/Vs, current on/off ratio ~ 10^4^, photoresponsivity (R ~ 74 AW^−1^ @ 82 mW cm^−2^), detectivity (D^*^ ~ 1.25 × 10^11^ Jones), external quantum efficiency (EQE ~ 173%) and rapid photoresponse (rise/fall time ~ 75/3 µs) as compared to individual ReSe_2_ device (mobility = 36 cm^2^ V^−1^s^−1^, I_on_/I_off_ ratio = 1.4 × 10^5^–1.8 × 10^5^, R = 11.2 AW^−1^, D* = 1.02 × 10^10^, EQE ~ 26.1%, rise/fall time = 2.37/5.03 s). Such remarkable performance is due to the combined result of strong light absorption of ReSe_2_ and high carrier transport of graphene. Moreover, low value of Schottky barrier height (SBH) for Gr/ReSe_2_-HS (9.02 meV @ V_bg_ = 40 V) confirms that graphene is somehow working as defects (due to Si/SiO_2_ dielectric substrate) suppressing layer. Furthermore, the HS was subjected to NO_2_ gas environment under various humidity conditions to test its aptitude for the gas sensor at room temperature (26.85 °C). The results demonstrated a high response, good reversibility, and gas selectivity under light irradiation of 532 nm. Interestingly, the proposed HS has illustrated an excellent response even toward low ppb-level NO_2_ exposure (20 ppb), revealing the proposed HS is superior to most of the reported literature. To conclude, our Gr/ReSe_2_-HS is capable of demonstrating excellent electro-optical as well as gas sensing performance simultaneously and, therefore, can be used as a building block to fabricate next-generation photodetectors and gas sensors to further enhance optoelectronics research domain and internet of things (IoT) devices. Moreover, it offers a potential sensing platform for cost-effective environmental monitoring systems.

## Data Availability

The data can be requested from corresponding author upon reasonsble request.

## Supplementary Materials

The following supporting information can be downloaded at: https://www.mdpi.com/article/10.3390/nano12213713/s1, Figure S1: Gr/ReSe_2_ heterostructure fabrication step-by-step detail; Figure S2: (a) AFM image (scale bar: 3 µm), and (b) corresponding height profile to accurately assess ReSe_2_ flake thickness; Figure S3: (a) Transfer characteristics (I_ds_–V_bg_) of mono-layer graphene measured at V_ds_ = 0.1 V reveals CNP = −8 V. (b) Output characteristics (I_ds_–V_ds_) calculated at various V_bg_ from −40 to 50 V reveal ohmic behavior of monolayer grapheme; Figure S4: Photocurrent measurement of ReSe_2_-based photodetector at mentioned conditions; Figure S5: MFC (mass flow controller) setup to test individual flakes (Gr, and ReSe_2_) and Gr/ReSe_2_ heterostructure-based NO_2_ gas sensing performance; Figure S6: Room temperature comparative analysis of Gas sensing responses from individual Gr (black), individual ReSe_2_ (blue), and Gr/ReSe_2_ heterostructure (red) for NO_2_ (200 ppb) under laser power of 310 mW cm^−2^ with a wavelength of 532 nm; Figure S7: Room temperature transient response of Gr/ReSe_2_ heterostructure for NO_2_ (200 ppb) under laser power of 310 mW cm^−2^ with a wavelength of 532 nm; Figure S8: (a) Response factor of Gr/ReSe_2_-HS for 200 ppm NO_2_ exposure under different values of relative humidity. (b) Corresponding column bar representation of the maximum value of response factor of Figure S8a.
