# Supplementary material for: Bias-Modified Schottky Barrier Height-Dependent Graphene/ReSe2 van der Waals Heterostructures for Excellent Photodetector and NO2 Gas Sensing Applications
Source: Nanomaterials (Basel). 2022 Oct 22;12(21):3713. doi: 10.3390/nano12213713 (PMC9658387; doi:10.3390/nano12213713)
Supplement: Supplementary file 1 [file nanomaterials-12-03713-s001.zip › nanomaterials-1989967-supplementary.pdf]

Supporting information

Bias-modified Schottky barrier height-dependent Graphene/$ReSe_2$ van der Waals heterostructures for excellent photodetector and NO_2 gas sensing applications

Ghazanfar Nazir[a,*], Adeela Rehman[b], Sajjad Hussain[a], Othman Hakami[c], Kwang Heo[a], Mohammed A. Amin[d], Muhammad Ikram[e], Supriya A. Patil[a], Muhammad Aizaz Ud Din[f]

[a]Department of Nanotechnology and Advanced Materials Engineering, Sejong University, Seoul, 05006, Republic of Korea

[b]Department of Mechanical Engineering, College of Engineering, Kyung Hee University, Yongin 17104, South Korea

[c]Department of Chemistry, Faculty of Science, Jazan University, Jazan, Saudi Arabia

[d]Department of Chemistry, College of Science, Taif University, P.O. Box 11099, Taif 21944, Saudi Arabia

[e]Solar Cell Application Research Lab, Department of Physics, Government College University Lahore, Lahore, 54000, Punjab, Pakistan

[f]School of Materials and Energy, Southwest University, Chongqing 400715, China

*Corresponding author: Dr. Ghazanfar Nazir

E-mail address: gnazir@sejong.ac.kr

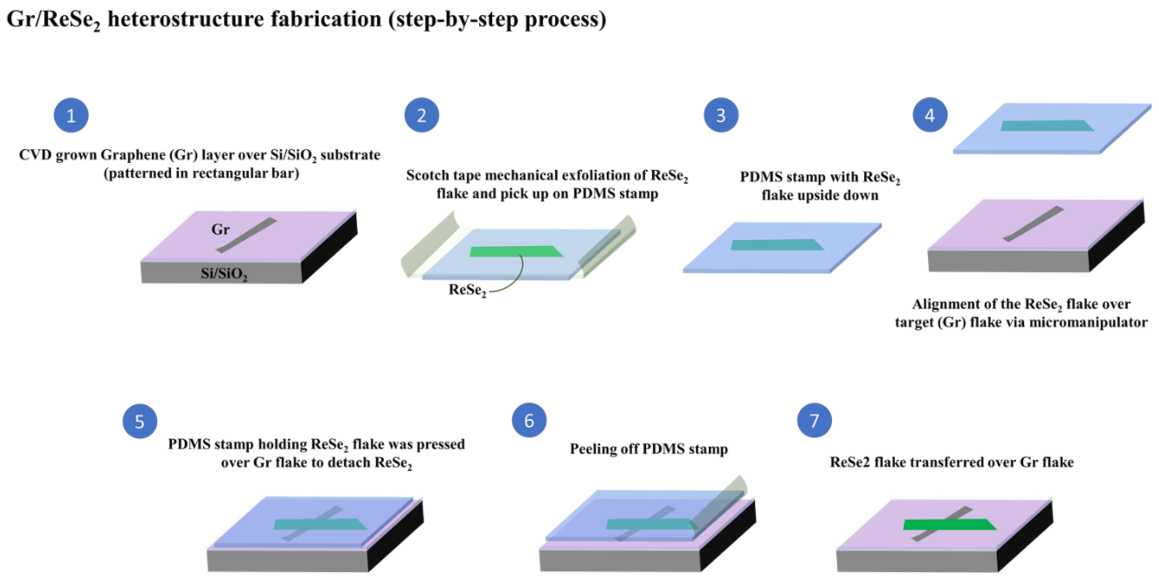

Fig. S1. Gr/$ReSe_2$ heterostructure fabrication step-by-step detail.

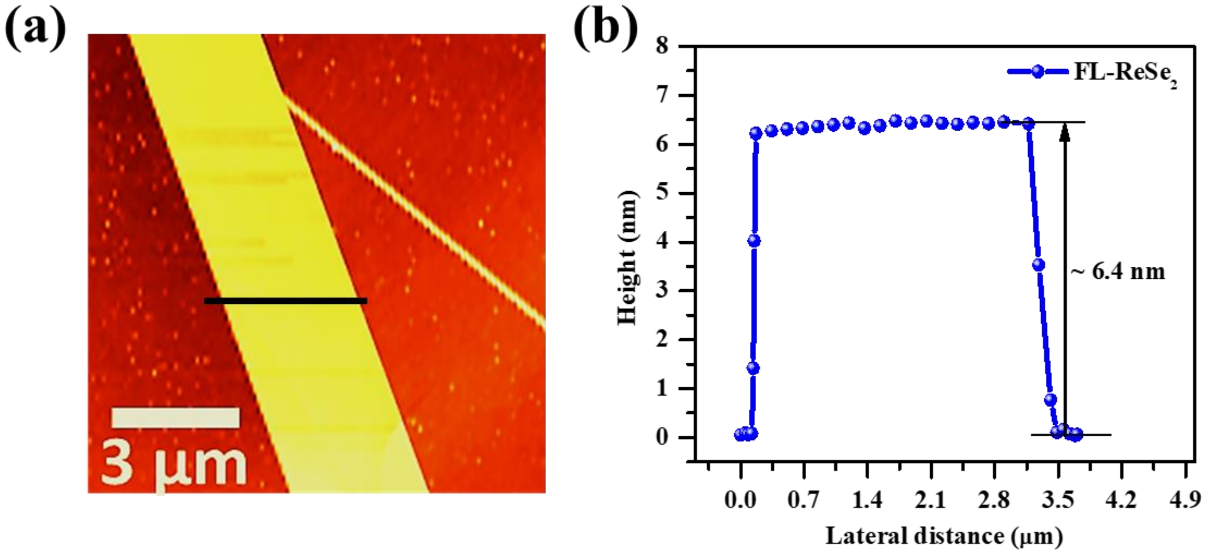

Fig. S2 (a) AFM image (scale bar: 3 µm), and (b) corresponding height profile to accurately assess $ReSe_2$ flake thickness.

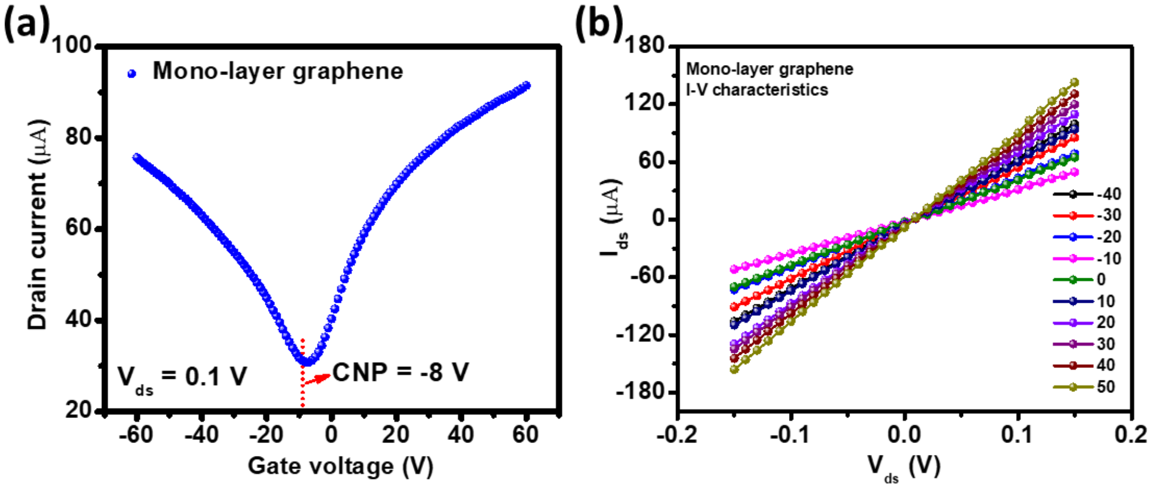

Figure S3. (a) Transfer characteristics (I_{ds} - V_{bg}) of mono-layer graphene measured at V_{ds} = 0.1 V reveals CNP = -8 V. (b) Output characteristics (I_{ds} -V_{ds}) calculated at various V_{bg} from -40 to 50 V reveal ohmic behavior of monolayer graphene.

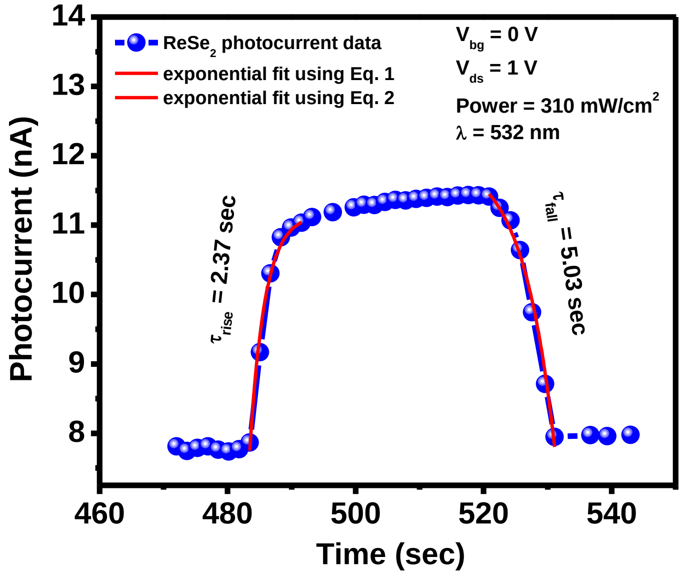

Fig. S4 Photocurrent measurement of $ReSe_2$-based photodetector at mentioned conditions.

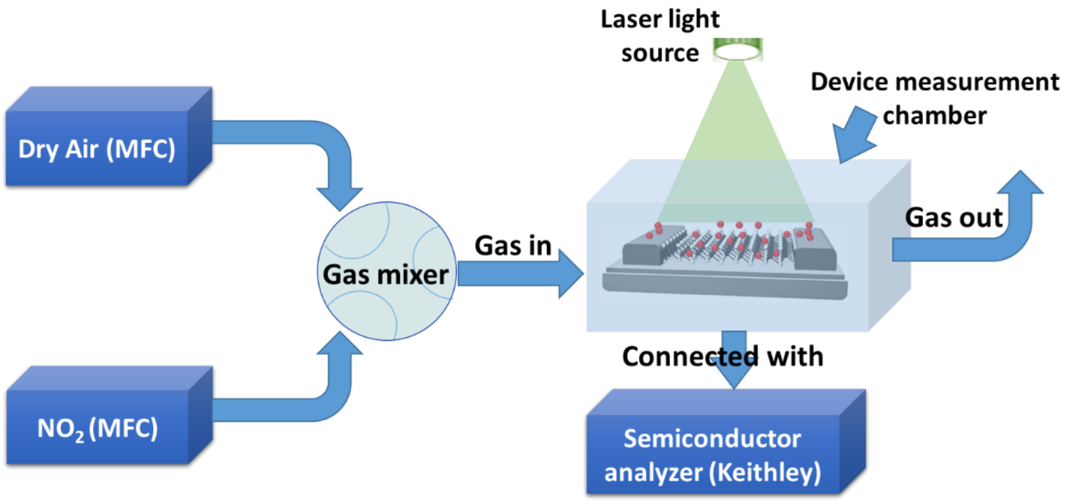

Fig. S5 MFC (mass flow controller) setup to test individual flakes (Gr, and $ReSe_2$) and Gr/$ReSe_2$ heterostructure-based NO_2 gas sensing performance.

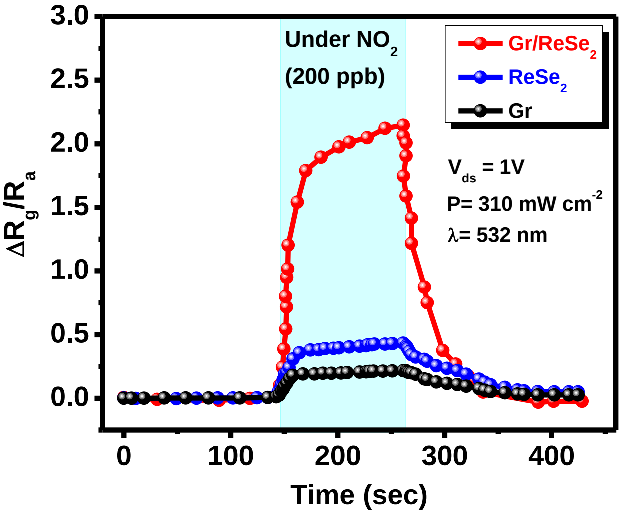

Fig. S6 Room temperature comparative analysis of Gas sensing responses from individual Gr (black), individual $ReSe_2$ (blue), and $Gr/ReSe_2$ heterostructure (red) for NO_2 (200 bb) under laser power of 310 mW cm^{-2} with a wavelength of 532 nm.

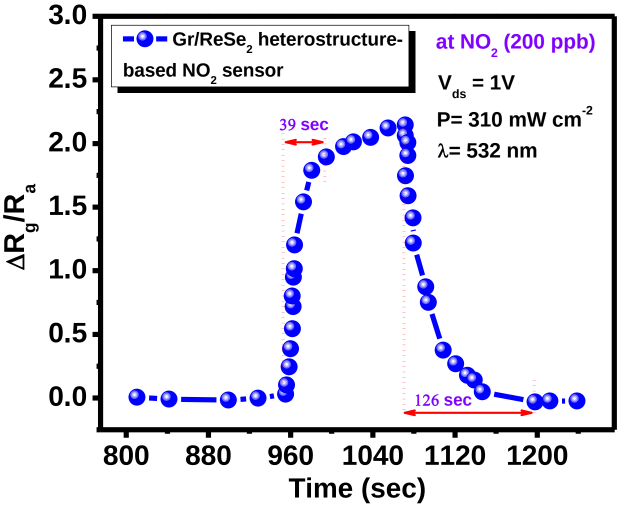

Fig. S7 Room temperature transient response of Gr/ReSe2 heterostructure for NO_2 (200 ppb) under laser power of 310 mW cm^{-2} with a wavelength of 532 nm.

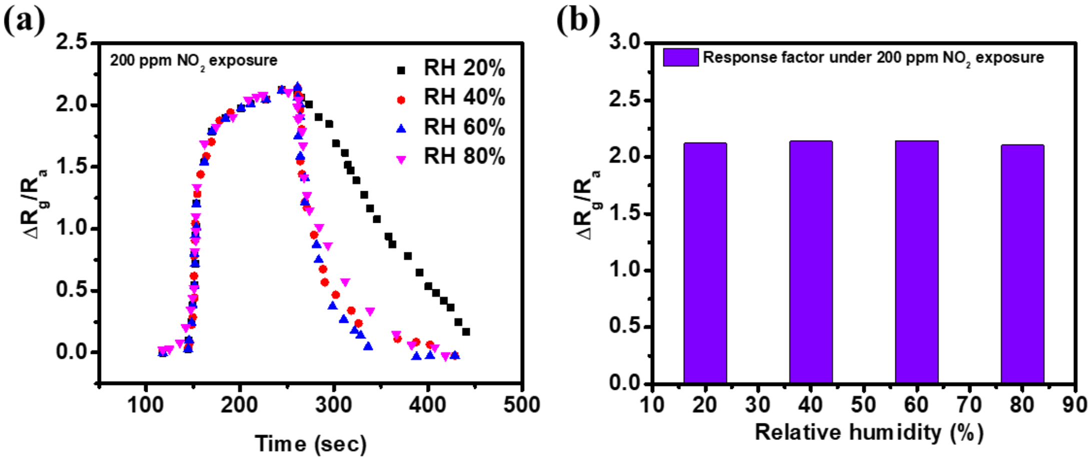

Fig. S8 (a) Response factor of Gr/$ReSe_2$-HS for 200 ppm NO_2 exposure under different values of relative humidity. (b) Corresponding column bar representation of the maximum value of response factor of Fig. (a).
